# Supplementary material for: From sequence to enzyme mechanism using multi-label machine learning
Source: BMC Bioinformatics. 2014 May 19;15:150. doi: 10.1186/1471-2105-15-150 (PMC4229970; doi:10.1186/1471-2105-15-150)
Supplement: Additional file 2 — Java code of ml2db. Additional file ml2db_code.tar.gz contains the Java source code to run the multi-label machine learning experiments and save the results to database. The code’s Javadoc is included. [file 1471-2105-15-150-S2.zip › additional file 2/ml2db/utils/doc/uk/ac/ed/inf/utils/database/class-use/TableColumn.html]

Uses of Class uk.ac.ed.inf.utils.database.TableColumn


---


|  |  |  |
| --- | --- | --- |
| **Overview** \| **Package** \| **Class** \| **Use** \| **Tree** \| **Deprecated** \| **Index** \| **Help** | |  |
| PREV   NEXT | **FRAMES**    **NO FRAMES**     **All Classes** |  |


---


## **Uses of Class uk.ac.ed.inf.utils.database.TableColumn**

| Packages that use TableColumn | |
| --- | --- |
| **uk.ac.ed.inf.utils.database** |  |

Uses of TableColumn in uk.ac.ed.inf.utils.database

| Methods in uk.ac.ed.inf.utils.database that return TableColumn | |
| --- | --- |
| `static TableColumn` | `DbUtils.getIdColumn()`             get the definition for an "id" column of type auto-increment (which is primary key) |
| `static TableColumn` | `DbUtils.getTimeDownloadedColumn()` |
| `static TableColumn` | `DbUtils.getTimestampColumn()` |

| Methods in uk.ac.ed.inf.utils.database that return types with arguments of type TableColumn | |
| --- | --- |
| `java.util.Vector<TableColumn>` | `Table.getColumns()`             Get the `TableColumn` list |

| Methods in uk.ac.ed.inf.utils.database with parameters of type TableColumn | |
| --- | --- |
| `int` | `TableColumn.compareTo(TableColumn f2)`             Compares two columns by their name, returns -1 if this column comes alphabetically before f2, 0 if the two are equal and +1 if f2 comes alphabetically before this column. |
| `boolean` | `TableColumn.equals(TableColumn anotherColumn)`             Compares two columns by their name, returns true if the two columns have the same name, false otherwise. |

| Method parameters in uk.ac.ed.inf.utils.database with type arguments of type TableColumn | |
| --- | --- |
| `boolean` | `DbCreator.generateTable(java.lang.String tableName, java.util.Vector<TableColumn> columns)` |
| `void` | `Table.initialise(java.lang.String tableName, java.util.Vector<TableColumn> columns)` |

Constructor parameters in uk.ac.ed.inf.utils.database with type arguments of type TableColumn

`Table(java.lang.String tableName,
java.util.Vector<TableColumn> columns)`
  
          List of primary key fields (starting from 0)

`Table(java.lang.String tableName,
java.util.Vector<TableColumn> columns,
DbManager dbManager)`

---


|  |  |  |
| --- | --- | --- |
| **Overview** \| **Package** \| **Class** \| **Use** \| **Tree** \| **Deprecated** \| **Index** \| **Help** | |  |
| PREV   NEXT | **FRAMES**    **NO FRAMES**     **All Classes** |  |


---
